# Quantification of STEM Images in High Resolution SEM for Segmented and Pixelated Detectors

**DOI:** 10.3390/nano12010071

**Published:** 2021-12-28

**Authors:** Ivo Konvalina, Aleš Paták, Martin Zouhar, Ilona Müllerová, Tomáš Fořt, Marek Unčovský, Eliška Materna Mikmeková

**Affiliations:** 1 Institute of Scientific Instruments of the Czech Academy of Sciences, Královopolská 147, 612 64 Brno, Czech Republic; patak@isibrno.cz (A.P.); zouharm@isibrno.cz (M.Z.); ilona.mullerova@isibrno.cz (I.M.); fortt@isibrno.cz (T.F.); eliska@isibrno.cz (E.M.M.); 2 Thermo Fisher Scientific Inc., Vlastimila Pecha 12, 627 00 Brno, Czech Republic; marek.uncovsky@thermofisher.com

**Keywords:** STEM segmented detector, pixelated detector, scanning electron microscopy, Monte Carlo simulations, ray tracing, quantitative imaging

## Abstract

The segmented semiconductor detectors for transmitted electrons in ultrahigh resolution scanning electron microscopes allow observing samples in various imaging modes. Typically, two standard modes of objective lens, with and without a magnetic field, differ by their resolution. If the beam deceleration mode is selected, then an electrostatic field around the sample is added. The trajectories of transmitted electrons are influenced by the fields below the sample. The goal of this paper is a quantification of measured images and theoretical study of the capability of the detector to collect signal electrons by its individual segments. Comparison of measured and ray-traced simulated data were difficult in the past. This motivated us to present a new method that enables better comparison of the two datasets at the cost of additional measurements, so-called calibration curves. Furthermore, we also analyze the measurements acquired using 2D pixel array detector (PAD) that provide a more detailed angular profile. We demonstrate that the radial profiles of STEM and/or 2D-PAD data are sensitive to material composition. Moreover, scattering processes are affected by thickness of the sample as well. Hence, comparing the two experimental and simulation data can help to estimate composition or the thickness of the sample.

## 1. Introduction

Scanning electron microscopes (SEMs) equipped with scanning transmission electron microscope (STEM) detectors offer unique imaging modes. STEM works similarly to SEM, the primary beam electrons scan in a raster pattern across the thin sample and the transmitted electrons (TEs) are generated. TEs have an energy and angular distribution depending on the energy of the primary electrons, the material and the thickness of the sample. TEs collected near the optical axes give the bright-field (BF) image. These electrons have either not been scattered at all, direct beam, or have been (in)elastically scattered through angles of milliradians or less. STEM also offers dark-field (DF) imaging [1]. The images are formed from either inelastically scattered electrons at larger angles or from elastically scattered electrons. A unique imaging mode is high angle annular dark field (HAADF) [2]. The HAADF signal depends on density and thickness of the sample and it is proportional to the nth power of the atomic number *Z* [3,4]. Deviations from this simple power law of (effective) *Z* have been reported for small detection angles and heavy elements [5].

Today’s scanning electron microscopes can operate in the “cathode lens” mode [6,7] which utilizes a high negative bias on the sample and a strong electric field around the sample. This mode offers a convenient tool for controlling the landing energy of electrons down to units of electronvolts. Moreover, the field accelerates and collimates the signal electrons to detectors above and below the sample, thereby accomplishing high collection efficiency and high amplification of the image signal [8,9,10]. An additional bonus in STEM imaging is the ability to collect secondary electrons (SEs) which are accelerated to the STEM detector. Slow electrons represent an efficient and powerful probe for study and characterization of samples [11,12]. They can even be used to remove polymer residues from graphene, and it is possible to determine the number of layers in few-layer graphene using low-energy electron reflectivities/transmissivities [13].

The original single-segment integral detectors [14] are being replaced by new types of angle-selective detectors [15], a segmented detector and a pixel array detector (PAD), respectively. Standard STEM detectors, which are formed of several segments for imaging in BF and DF modes, consist of many photodiodes. This detector has a wide range of applications, for example, in material analysis [16,17,18,19], in life science [20] and in semiconductors [21]. Conversely, the full annular distribution is not resolved because the signal incident on the inactive areas between the individual segments is lost. The PADs enable collecting electron diffraction patterns for backscattered electrons and TEs. Acquisition of electron backscattered diffraction (EBSD) patterns above the sample allows to perform tilt-free EBSD [22]; large EBSD mapping is possible if one uses a detector that consists of several PADs sensors [23]. The PADs can also be designed for diffractive STEM applications [24]. STEM using a pixelated detector complements and increasingly replaces existing imaging approaches. PAD provides access to additional detailed information on strain, orientation, electric or magnetic fields. It even potentially allows for resolution enhancement via correction of aberrations [25] or Ptychography methods [26]. However, at present, the speed of these detectors is lower than the one of segmented detectors or detectors typically equipped with a photomultiplier.

In order to comprehend the image contrast formation in the above described different microscopic modes, a deeper understanding of these regimes is required and computer simulations are necessary. Since the Ulam’s invention and first von Neumann’s implementation of the Monte Carlo (MC) method, this simulation approach (for an introduction see [27,28]) spread over several scientific branches such as high-energy particle physics and astrophysics [29] or medical physics [30] and electron microscopy, see [31,32,33,34] and references therein. We have decided to simulate the passage of electrons through matter by employing Geant4 [35] open source platform with a wide user base and several modules usable for lower primary electron energies (up to tens of keV) in SEM and also a package [36] with a good description of even lower signal electron energies. This is necessary because SEs with energies below 50 eV contribute to the image contrast. Microscopists can access quantities such as scattered electron energy angular distribution, not attainable directly in their SEM experiments, via MC simulation.

Trajectories of TEs are influenced by the fields between the sample and the detector. The propagation of electrons in a region of space where they are exposed to a magnetic or electrostatic field or both cannot be easily estimated. Ray-tracing programs can be used to analyze the impact position of electrons on individual parts of the detector, so-called acceptance diagrams.

Physical interpretation of the experimental data acquired using a segmented detector, more precisely a group of a detector and related amplifiers, or its comparison with simulations may not always be straightforward. See, e.g., [37], and references therein for a recent overview of quantitative STEM with segmented detectors.

It is known that sensitivity of some of the detectors varies from one segment to another [38], and this can be handled by measuring using the bright field (BF) segment only and repositioning it in the detector plane. An overall characterization of variation of the response has already been proposed [39]. Furthermore, it was reported that response of the annular dark-field (ADF) detector may vary in different areas [40]. The paper shows that response w.r.t. beam current starts with an offset, is linear, and it finally reaches a saturation for sufficiently high beam current. Non-linear effects for beam current are also reflected in non-linear dependence of response on current and dwell time for current higher than 0.5 pA [41]. Therefore, it is desirable to compare sets of measurements with their settings “sufficiently close”. The usual contrast calculation/calibration uses dark current (DC) and full signal on detector without sample [42]. This approach is too restricting; one has to use low current (not to oversaturate the detector or its segment during the full signal measurement, then sample measurement is too noisy) or low contrast (sample measurement exhibits too small dynamic range). Ray-traced MC simulations provide different quantities—counts of electrons (on a given segment)—and corresponding energy dose. We show that none of them lead to a reliable comparison with the segmented STEM experiments. The new quantitative method presented in this paper was designed with the different responses of individual segments in mind and it is more elaborate than the aforementioned contrast calibration. We apply it in the case of two different amorphous samples, low atomic number carbon (commercially available) and high atomic number in house magnetron sputtered molybdenum thin nano-films. However, comparing the energy dose directly with the 2D-PAD data gives a sound agreement.

The data were acquired using two detection systems for TEs in Thermo Fisher Scientific [43] microscopes (Thermo Fisher Scientific Brno Ltd., Brno, Czech Republic). The first is a segmented semiconductor STEM detector in a Magellan 400 FEG microscope. The second is the pixel array detector-T-pix in a Helios microscope.

## 2. Materials and Methods

We describe the imaging modes, detectors, the measurements and materials studied. The theoretical part deals with the interaction of electrons with matter and with the ray-tracing of the TEs in an electrostatic and/or magnetic field in the space between the sample and the detectors. It contains a detailed description of the data processing, including our calibration curve method.

### 2.1. Microscope Imaging Modes

The SEMs we used allow observing samples in four imaging modes. Two modes of objective lens, namely high resolution (HR) and ultra-high resolution (UHR), differ by their resolution and by the presence or absence of a magnetic field around the sample [9,44]. If the beam deceleration (BD) mode is chosen, then an electrostatic field around the sample is added and two further microscope modes HR+BD and UHR+BD, become available.

In HR and HR + BD modes, the sample and its surroundings are free of magnetic fields. The electrostatic field between the sample and the detector (HR + BD mode) collimates TEs toward the optical axis. We can detect signal of TEs scattered to large polar angles with respect to the optical axis.

**Figure 1:**
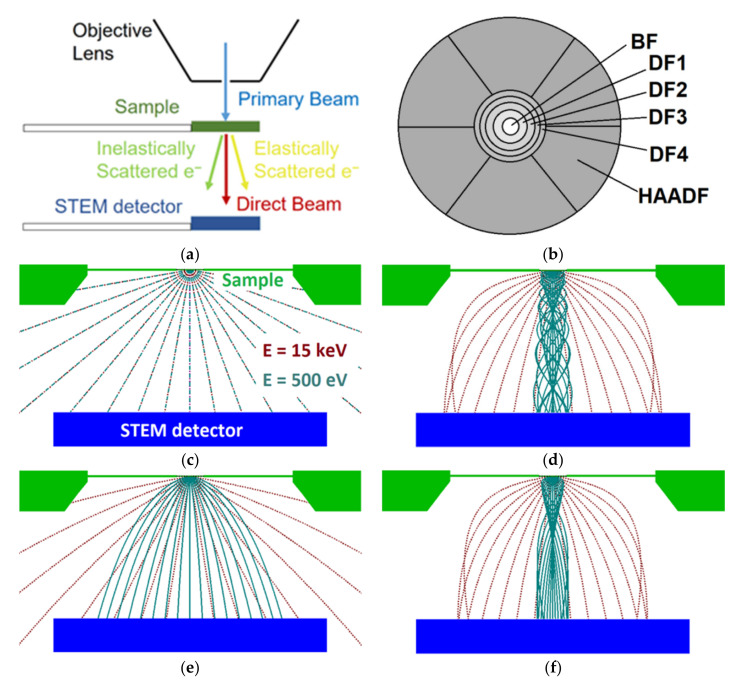
Scheme of the STEM detector and trajectory simulations for microscopic modes: (**a**) Simulated arrangement (side view); (**b**) STEM detector—segments (top view); simulated profiles of radial coordinate *r* of selected trajectories (polar angle from 0 to 90 deg with step 10 deg, two different energies: 15 keV red, 0.5 keV green) along the optical axis in the case of four available modes: (**c**) HR; (**d**) UHR; (**e**) HR + BD; (**f**) UHR + BD. The sample detector distance is 12.5 mm, and the potential at the sample is −4 kV if BD mode is active and 0 V if not. The magnetic field is implicitly determined such that the beam is focused at the sample in the (corresponding) working distance of 4.5 mm.

The sample is placed in a strong magnetic field in the case of both UHR and UHR + BD modes. The electrons have spiral trajectories because of the magnetic field and they can cross an arbitrary but fixed optical axis plane, e.g., defined by optical axis and initial non-central velocity, several times before they reach the detector. See Figure 1d,f for simulations of selected trajectories at two different energies. The extension of the displayed *r*(*z*) sections to 3D must include spiraling with Larmor frequency. Thus, we see the angular distribution is not simply interpretable as in the case of the HR mode, i.e., Figure 1c,e.

TEs and their trajectories are studied with respect to their angular and energy distribution in each mode of the microscope. The TEs that reach the detector are analyzed in detail in this work.

### 2.2. STEM Segmented Detector Measurement

The basic layout scheme of the objective lens, sample holder and STEM detector in the microscope is shown in Figure 1a.

The STEM detector is a semiconductor detector located below the sample and it detects TEs. It consists of six concentric segments, the central (BF), four annuli (DF1-DF4) and the outer largest segment of HAADF, which is divided into six segments (Figure 1b).

**Figure 2:**
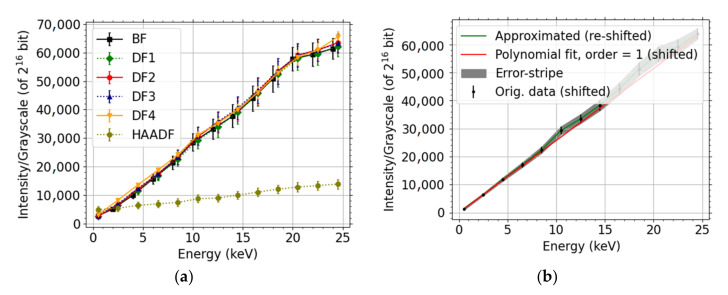
Calibration curves for the segments of detector. (Contrast DB, a.k.a. gain, equal to 83%, beam current = 1.56 pA, dwell time = 10 μs) (**a**) as were, (**b**) CC in the case of DF4, with DL subtracted, and a polynomial fit of a sufficient number of values close to *E* = 0 eV used to estimate the DL.

In order to be able to quantify the experimental data, it is necessary to perform measurements under the same defined conditions and calibrate the detector. For that reason, an additional series of measurements without sample was performed. One series at a given segment of the detector *g_segment_* consists of measurements at fixed conditions, except for “incident” energies, see Figure 2a. This series *g_segment_(E)* provides intensity *I* as a function of incident energy of detected electrons, the dependence being fairly linear. We shall drop the subscript segment in the following for the sake of brevity; it will be restored only when explicitly needed. The collection of series at given conditions for all segments provides a set of calibration curves (CCs) for a measurement at the given conditions. The CCs, without samples, were acquired at a lower current in order to avoid oversaturation of the detector. Using a different current is not a problem in the below described data processing, provided that the detector response is a suitable (e.g., linear) function of the current to a sufficient degree within the current range used. The details will be discussed in Section 2.5.

The detector has been gradually retracted to measure CCs of all segments. The retraction is possible in a service mode. Then, the detector was recentered. We estimate the centering precision to be better than 50 μm. In addition, a blanked beam measurement was performed to estimate the dark current (DC). The measurement with a fixed energy step was automated using IFAST.

Let us define dark-level (DL) corresponding to a given CC as $b=\underset{E\to 0}{\lim}g\left(E\right)$. Determination of DL is performed using polynomial extrapolation. Example of CC with DL subtracted is displayed in Figure 2b.

We verified explicitly that the values of DC and DL are practically the same. Both limits of zero-current (DC) and zero-energy (DL) correspond to no incident electrons on the detector. Hence the fact that they are the same does not come as a surprise.

We apply the above described calibration method to relative signals, thus avoiding the need of recalculating the data per electron which removes the necessity to measure the incident beam current.

### 2.3. 2D-PAD Measured Data and Their Processing

**Figure 3:**
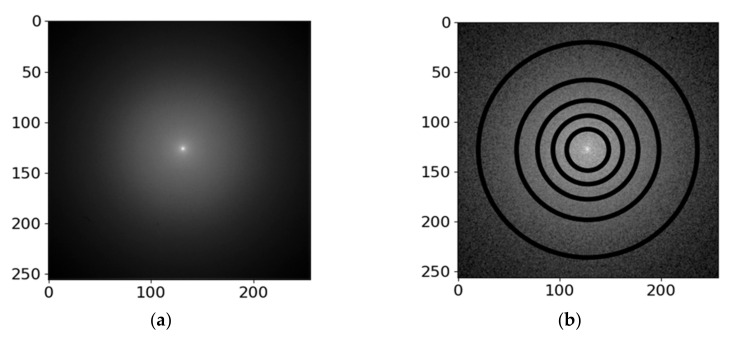
C 100 nm at 15 keV (**a**) 2D-PAD diffractogram, (**b**) simulated distribution of energy dose (glassy-carbon) in the 2D-PAD plane with the appropriately rescaled inactive areas of the segmented STEM detector up to HAADF indicated. The contrast of both data has been enhanced by the gamma correction, value 𝛤 = 0.5.

The measurement using the pixelated detector is fast and no scanning over the sample area is performed, hence the surface diffusion of adsorbed hydrocarbon contaminants is expected to be negligible. The contamination will be further discussed in Section 2.6. The square 2D-PAD detector has pixels of size 0.055 mm, and the dimensions are 256 × 256 pixels. The diffractogram is collected from a single spot on the sample. An example of the measured diffractogram is presented in Figure 3a. We have also displayed corresponding simulation results (obtained using a method yet to be described) explicitly marking inactive areas between the individual segments of the segmented STEM detector in Figure 3b. This illustrates the advantage of the 2D-PAD over segmented STEM detectors—no signal is lost within the boundaries of the advanced detector and it provides richer angular information when compared to the segment-integrated signal.

Since the measured samples, thin foils, are assumed to be homogeneous and the electrons propagate in fields that are axially symmetric, the measured intensity should inherit its symmetry from the fields. Hence, it is meaningful to display radial or polar angle dependence of the intensity. The position of the centre is estimated using an intensity-weighted average of positions. Then, we slice the 2D-PAD by a series of equidistant concentric annuli. We use the total energy dose from the fully contained pixels, corresponding count denoted by *N_px_*, and multiply it by a correction factor *c_area_*. The factor *c_area_* is equal to the geometrical area of each annulus *S_annulus_* divided by the area of these pixels *N_px_S*_1 *px*_. This correction of the data to “full-area” is relevant at larger radii/angles where the annulus is not fully enclosed in the 2D-PAD. It enables to display a larger range with sufficient reliability, except for the outermost incomplete yet partially contained annuli.

Error, corresponding to a value obtained within an annulus, is estimated as follows. We use standard deviation. The correspondence is fully achieved by multiplying by, in general, non-integer “corrected pixel count” *S_annulus_*/*S*_1 *px*_. Step in radius corresponds to one pixel but the annuli width is doubled to produce a somewhat smeared-out value.

We performed dark-current measurements with the beam blanked. They indicate that both the dark current and its error are negligible (mean value 0.3 and corresponding standard deviation equal to 3), hence they are not included in the considerations. In order to avoid possible edge effects, we exclude the outermost rows and columns on all sides of 2D-PAD.

### 2.4. MC Simulations

Simulation of the scattering provides electrons outgoing from the sample and it can be viewed as both energy and spatially resolved data in contrast to the cumulative experimental data in standard STEM imaging. Therefore, simulation may provide valuable insight into the observed image. The frequently used tools, describing amorphous materials well, are based on Monte Carlo (MC) methods. MC utilizes a random numbers generator for a large number of electron trajectories. We note that MC is not free from approximations and simplifications. It is inherently a statistical method; hence, it is desirable to analyze errors of the simulation too. We perform the statistical analysis to estimate errors of the simulations by splitting the MC data into 10 batches and calculating corresponding standard deviation. The details will be described in the next section. Furthermore, if the de Broglie wavelength of electrons is comparable to the distances of scattering centers, interference effects can start to play a role, especially for crystalline samples, and another approach is preferable—a multislice algorithm and/or density functional theory.

MC is well suited to simulate the thin non-crystalline foils used in the present study. Electron scattering simulations were performed using the MC method as implemented in Geant4 [35]. The range of reliable applicability of existing standard modules such as PENELOPE-2006 [45], Livermore [46] is limited from below by approximately 250 eV. In order to describe the SEs in a better way, we utilized the module “low-voltage SEM” [36]. This module provides a good description of electrons even below 50 eV. The simulations provide both positions and velocities of the electrons outgoing from the sample, i.e., initial conditions for ray tracing below and/or above the sample. Mass densities, in g/cm^3^, used for the materials are 1.47 for C—glassy-carbon, 2.27 for C—graphite, and 10.28 for Mo.

### 2.5. Processing of MC Data

These intermediate results of MC simulations, initial positions and velocities of outgoing electrons serve as an input into tracing program Electron Optical Design (EOD) [47] which produces distribution of electrons and their energies in the detector plane using the finite element method.

For the selected material, of a given thickness, and the landing energy of the primary electrons reaching the sample, we simulate the spectrum of TEs. Each ray has its initial parameters with which the corresponding electron leaves the lower plane of the sample. The electron trajectory is determined by its initial position, energy and initial azimuthal and polar angle of its velocity. We have used the tracing software to propagate electrons, corresponding to a given MC simulation for a given sample, from below the sample to the detector plane. In order to be able to achieve this, the exact magnetic and electrostatic field must be well known. This implies that it is crucial to know the real geometry of all parts and components in a simulated arrangement.

Initially, fifty thousand of sample-incident electrons were traced. Increasing the amount of electrons to one million provides a better comparison with angle-resolved 2D-PAD measurements. Because the tracing is time-expensive, the 10^6^ electrons were propagated as in a field-free environment. This corresponds well with the fact that all the 2D-PAD measurements were performed in the HR regime. The resulting distribution has been divided into the individual concentric segments of the STEM detector thus yielding intensity/histogram data corresponding to the simulations. Furthermore, detailed knowledge of the energy of the individual particles allows us to split the intensity into forescattered electrons (FSEs) and SEs (energy up to 50 eV). This will demonstrate that the simulations predict a non-negligible contribution of the SEs into the intensities recorded by the segmented detector in the case of BD-modes.

The distribution of energy dose from freely propagated electrons is divided into individual pixels in the 2D-PAD plane. The values forming the theoretical angular profiles are obtained, from these pixel-resolved data, in the same manner as the 2D-PAD data discussed in Section 2.3.

The point of our data-processing method applied to the segmented detector is that the detector does not measure simple counts *S*(*E*) of detections but it is also sensitive to the incident energy *E*. This core assumption is clearly confirmed in Figure 2 showing example CCs-the gray-scale *g*(*E*) at fixed current depends on the electron energy.

Because of that, intensity/gray-scale *G* depends on incident energy *E* of a detected electron, through corresponding CC factor *g*(*E*), and the count of electrons *S*(*E*) at the given energy. We postulate the following relation

(1) $$G=\int dE\tilde{g}\left(E\right)S\left(E\right),$$

where $\tilde{g}\left(E\right)\;=g\left(E\right)-b$ is DL-corrected CC.

**Figure 4:**
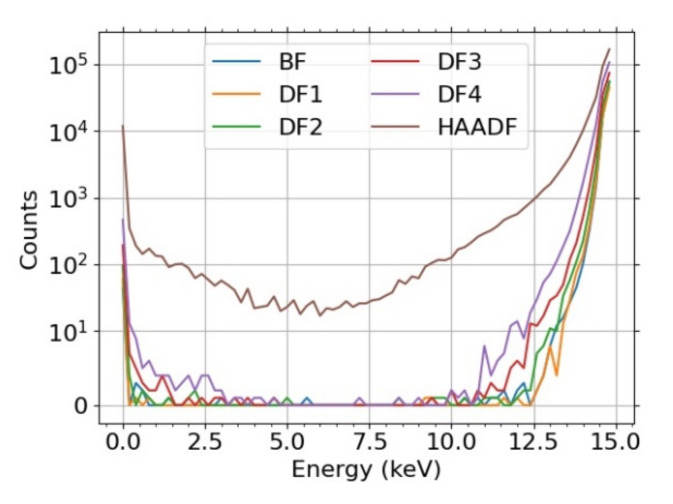
C 100 nm, 15 keV, glassy-carbon, MC for 10^6^ electrons. The visualization uses a symlog scale with the linear threshold equal to 10.

The reader can familiarize with the above equation by considering two simple examples. Number one, uniform distribution, i.e., *S*(*E*) = constant corresponding to measuring a CC. Number two, weighted sum of δ-distributions for few different energies. A more realistic situation corresponding to MC simulations of glassy-carbon at 15 keV is presented in Figure 4. Integral expressions for total intensity relating electron flux and detector response already exist, see e.g., [37,48]. The latter reference presents a non-trivial dependence of detector response on incident (i.e., sample-scattering) angle which we do not consider. Instead, we consider energy dependence that is much more important in our setup than the incident angle. To the best of our knowledge, the aforementioned integral method has not been applied to the energy of the incident beam yet.

The Equation (1) implicitly assumes appropriate dependence on current (directly related to *S*), already mentioned in Section 2.2. The meaning of *G* on the left-hand side of the Equation (1) is two-fold. First, it corresponds to the DC-corrected measured intensity *I*, i.e., *I* − *b*. Second, the whole relation provides a prescription for converting the traced MC results *S*(*E*) to grayscale.

The scale of the measured data and thus converted MC results may still differ. This is due to different “in-flux” of particles, i.e., current. In order to normalize both data-sets to the same current precisely, one would have to independently verify the current used in the measurements. We circumvent this inconvenience by comparing relative—with respect to a reference segment—grayscales.

This is similar to contrast calculations $C=\frac{I-I_{DC}}{I_{max}-I_{DC}}$ with maximum value replaced by the experimental grayscale at the reference segment. The DL must indeed be subtracted here in order to make such processing meaningful. Expressed as an equation, the analogy and comparison method can be written as

(2) $$\frac{I_{segment}-b_{segment}}{\underset{Experiment}{\underbrace{I_{ref.segment}-b_{ref.segment}}}}=I_{relative}\approx G_{relative}=\frac{G_{segment}}{\underset{MC\;via\;Equation\;\left(1\right)}{\underbrace{G_{ref.segment}}}}.$$

The DL value *b* can be subtracted from the measured data because the measurement conditions were the same apart from the current.

Let us illustrate that one can mix different currents among a series of measurements as long as the DL-corrected response CC function $\tilde{g}$ as a function of current *j* behaves as follows $\tilde{g}\left(n*j\right)=h\left(n\right)*\tilde{g}\left(j\right)$ where *h* is an appropriate function. E.g., it will apply to $\tilde{g}$ being a linear function or a homogeneous function. Consider lower current *j_c_*, that avoids oversaturation when performing CC measurement, and *n*-times higher current *j_f_* ensuring enough signal at the detector in the case of thin foils. Furthermore, if we assume the sample response to current is linear, then the corresponding electron counts are related by *S_f_* = *nS_c_*. The example CC data in Figure 2 are almost linear functions of energy for all segments, i.e., *g*(*E*) = *b* + *cE*. Let us note that *b* is independent of the current, i.e., $b{|}_{j=j_{f}}=b{|}_{j=j_{c}}$. Normalizing the grayscale per segment *k* leads to

(3) $$\frac{G_{i}}{G_{k}}=\frac{c_{i}}{c_{k}}\frac{\int dES_{i}\left(E\right)E}{\int dES_{k}\left(E\right)E}=\frac{c_{i}}{c_{k}}\frac{\varepsilon_{i}}{\varepsilon_{k}}$$

where $\varepsilon_{i}∶=\int dES_{i}\left(E\right)E$ denotes the total energy dose per *i*-th segment. The *S_i_* and *S_k_*, from a single series of measurements at the same current, appear in the integrands in a ratio. Furthermore, integral is linear. Therefore, the multiplicative factor determined by *n* cancels out in *G_i_*/*G_k_* if we substitute both *c* and *S* by values corresponding to measurements at the *n*-times higher current *j_f_*.

All the STEM measurements, including the CCs, contain fluctuations of intensity in the image. Statistical analysis of these fluctuations provides error for these measurements. Furthermore, the estimate of error-bars in the simulation is based on a single contribution from the MC simulations from splitting of the outgoing trajectories into 10 batches and performing statistical analysis of the results across the batches. Different processing approaches were tested in order to estimate the simulation errors. The selected approach uses histogramized angular profiles of these ray-traced batches and the value of error is equal to standard deviation. Each segment corresponds to one bin in the case of segmented STEM measurements. Since the 2D-PAD data will display comparison with different thicknesses, overlapping of the corresponding error stripes would make the images comprehensive on one hand and also unnecessarily complicated on the other hand. For that reason, we do not present the error stripes in this case and we merely state that they are comparable with the experimental ones provided they are obtained using the selected approach.

The same processing of the MC simulations, as in the case of experimental 2D-PAD data, leads either to large errors (non-batched) or oscillations in the average value (even for larger bin-sizes of batched histograms) indicating a million of particles is not enough for this approach in the case of molybdenum. Attempting to “homogenize” the pixelated sparse MC energy-dose data by ignoring pixels with almost vanishing energy-dose (less than 50 eV) decreased the width of error stripes but it still was not satisfactory.

Custom Python3 scripts [49,50,51] handle the data processing and visualization.

### 2.6. Samples

The new quantitative method is presented on two different amorphous samples, light element carbon and heavy element molybdenum thin films. Of course, the real samples are not truly amorphous and they contain crystalline grains. Unfortunately, the crystallography is not included in the Monte Carlo simulations and for the experiments the amorphous samples have to be prepared. The commercially available carbons films were provided by Lebow company [52] (100 nm thick films). The guaranteed thickness tolerance is equal to ±10% as standard. Molybdenum thin films of nominal thickness 110 nm were prepared by magnetron sputtering at the Institute of Scientific Instruments. The thickness has been independently verified using the profilometer at two different places yielding 108 and 113 nm.

The cleanness of the samples is important. Before each segmented STEM measurement, the samples were heated at 250 °C for 20 min to desorb hydrocarbons causing contamination, and after that immediately inserted to the microscope chamber, through load-lock, with subsequent cooling in there. We used every available method to reduce the effect of contamination as much as possible. The major source of the contamination is a surface diffusion of already present contaminants to the scanned area that results into cross-linked hydrocarbons. Moreover, the “standard” vacuum and possible additional impurities in the microscope chamber may result in an additional contamination that is deposited on the sample. In the case of STEM measurements, we always applied cryo-can—a can outside of the chamber which is filled with liquid nitrogen. The can is connected to the outside of the chamber resulting in a chamber wall being cooled down and some of the “pollutants” in the standard vacuum are deposited on the cooled part thus improving the quality of the vacuum with the effect lasting for several hours. The sample treatment was different in the case of the 2D-PAD measurements. Because the current prototype of the holder of the pixelated detector is not compatible with load-lock yet, the ex-situ heating is not beneficial. Therefore, it was omitted. The cryo-can was not applied as well.

## 3. Results

The two materials, carbon and molybdenum, that we analyzed with a STEM detector have nominal thicknesses of 100 nm (C) and 110 nm (Mo) and for that reason we used a landing energy of 15 keV, at which we have a sufficient signal of TEs. In the BD mode, we used the maximum possible bias voltage on the sample, i.e., 4 kV. The energy of the primary beam in the BD mode was thus 19 keV with the value of the landing energy still equal to 15 keV. In the case of 2D-PAD, we used an additional value of landing energy equal to 30 keV.

### 3.1. STEM Detector

**Figure 5:**
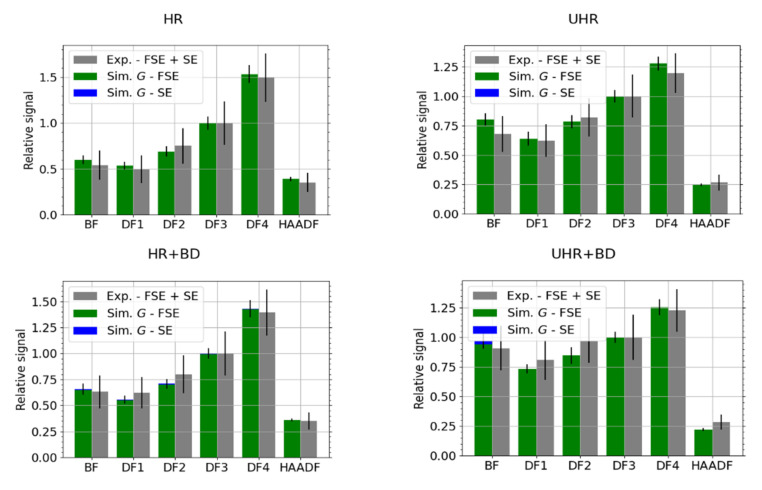
C—comparison of simulated and experimental data for HR, HR + BD, UHR and UHR + BD microscope modes, *E_L_* = 15 keV. The data have been scaled to the segment DF3. Simulation data are split into secondary electrons (SEs) and forescattered electrons (FSE).

Figure 5 shows a comparison of the simulated and experimental relative signal in individual segments of the STEM detector. The contribution of SEs to the total signal is evident only in the BF segment in the UHR + BD mode. The data in the graphs are normalized to the segment DF3.

The primary current was set to 6.3 pA. We measured the images from the individual segments of the detector with the same setting of the detection channel. This is a necessary condition for us to be able to compare the signal from the individual segments and then compare the measured data with the simulation results. The detection channel gain was set at 83% for all measurements of the carbon sample.

**Figure 6:**
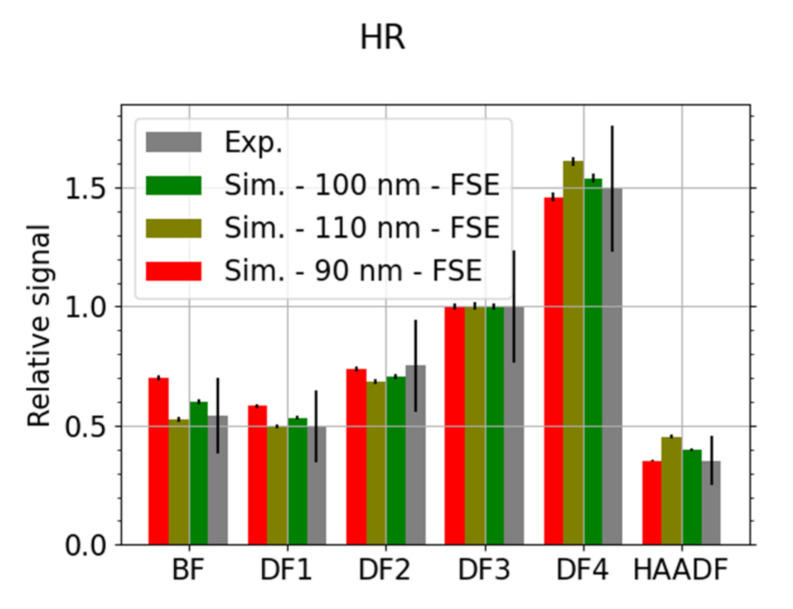
C—comparison of three simulated thicknesses and experimental data for HR microscope mode, *E_L_* = 15 keV. The data have been scaled to the segment DF3 and the MC simulated electrons, corresponding to 10^6^ incident particles, were freely propagated to the segmented detector. Contribution of the SEs is marginal in the HR mode.

The thickness of the sample affects the energy and angular distribution of the TEs. This causes a change in the signal intensity in the individual segments of the detector. Figure 6 is a comparison of experimental data with simulations for a nominal carbon sample thickness of 100 nm and for sample thickness limits given by a 10% tolerance. Different thickness values result in the signal being redistributed among the segments of the detector.

**Figure 7:**
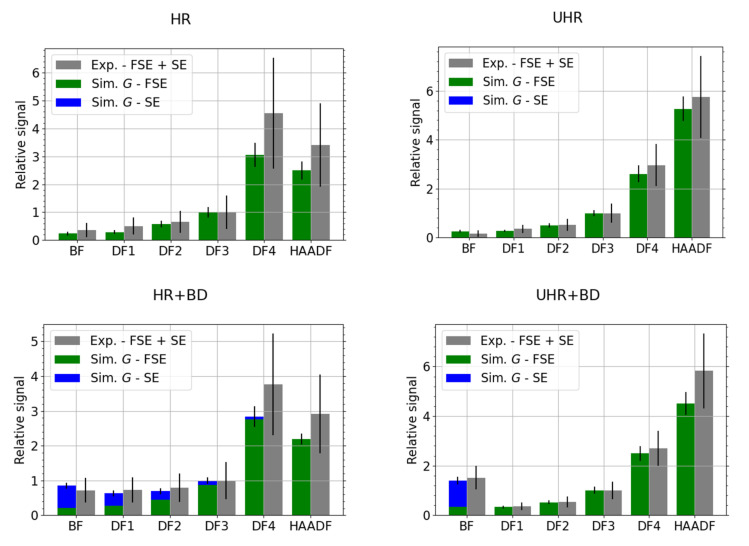
Mo—comparison of best-match simulated thickness 120 nm and experimental data for HR, HR + BD, UHR and UHR + BD microscope modes, *E_L_* = 15 keV. The data have been scaled to the segment DF3.

Due to the lower permeability of molybdenum than carbon, we chose a higher primary current, *I* = 13 pA. The detection channel gain in HR and HR + BD mode was 90.0%. Otherwise, the other parameters of the experiment were the same as when measuring the carbon sample. The results for molybdenum are presented in Figure 7. When compared with the results for the carbon sample, see Figure 5, we see that the BD modes collect more SEs. We have selected best-match thickness 120 nm for the MC simulations instead of the nominal value 110 nm (results not presented), as it provides a better agreement with the experimental data, especially in the HAADF segment.

### 3.2. 2D-PAD Detector

**Figure 8:**
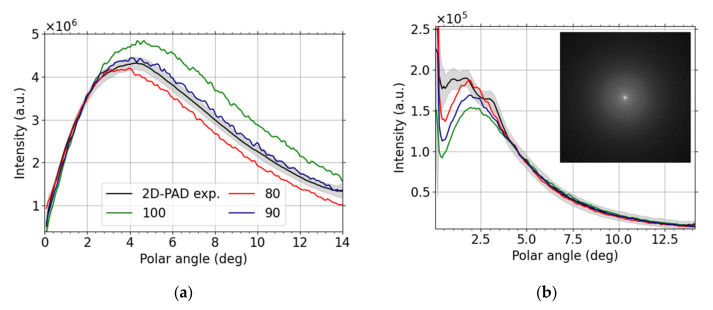
C, (**a**) 15 keV and (**b**) 30 keV, experimental (black with gray error stripe) and theoretical results for different thicknesses based on glassy-carbon MC simulations with 10^6^ particles freely propagated to the 2D-PAD plane. The inset shows the corresponding diffractogram with the Debye–Scherrer rings (contrast enhanced by the gamma correction, value Γ = 0.3, the camera length is 39.1 mm).

We present results obtained by the sophisticated modern pixelated detector in this subsection. The primary beam current was always set to 3.1 pA and the camera length varied according to working distance (the latter ranging from 3.5 to 4.9 mm). We plot the intensity as a function of the scattering polar angle for landing energy 15 keV and 30 keV in the case of carbon thin foil in Figure 8. We immediately see that the information we obtain from the 2D-PAD is much richer than from the standard STEM detector displayed in Figure 5. The angular resolution is much higher and it is possible to study differential cross-section in greater detail with 2D-PAD in SEM. We compare the measured data with the MC simulations for different thicknesses. The 15 keV results, Figure 8a, provides 90 nm simulations as the best match thickness. This analysis allows us to estimate local thickness of thin foils using non-destructive measurements. The 30 keV comparison, Figure 8b, shows a good agreement for polar angles larger than 4 degrees. We see oscillations for lower values of polar angles and the reason for the peaks to appear in this angular region is diffraction—the intensity from diffusive rings in the diffractograms sum up to form the mentioned peaks.

**Figure 9:**
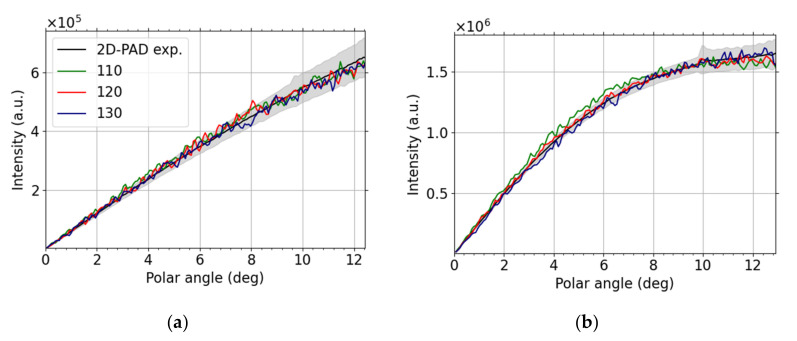
Mo, (**a**) 15 keV and (**b**) 30 keV, experimental (black with gray error stripe) and theoretical results for different thicknesses based on MC simulations with 10^6^ particles freely propagated to the 2D-PAD plane.

We present an analogous comparison for Molybdenum thin foil in Figure 9a (LE = 15 keV) and Figure 9b (LE = 30 keV). The resulting local thickness is 120 nm, determined from Figure 9b, for this foil composed of the heavier element. The higher landing energy is better suited for distinguishing the best-match thickness in this case. Moreover, no diffraction peaks are visible for the higher landing energy.

## 4. Discussion

We start the discussion by noting that the properties of thin foils and thin films depend significantly on preparation conditions. In the case of the amorphous carbon, the deposition energies affect the richness of sp^2^ and sp^3^ sites. The density increases with the fraction of the sp^3^ bonding, from 1.5 (0%) to 2.9 g/cm^3^ (80%) [53] Ab initio modelling leads to densities from 0.95 up to 3.5 g/cm^3^ [54]. The glassy-carbon MC simulations provide an excellent match to the measured carbon thin foil. Its density is at the lower range of the density interval discussed above. The match of a rather low density used in simulations means high content of sp^2^ sites in the measured sample. We have also tested MC simulations for higher density graphite carbon as an alternative. The lower density glassy-carbon data resulted in much better agreement of the best match thickness to the nominal thickness (hence we have presented only the glassy-carbon simulations).

**Figure 10:**
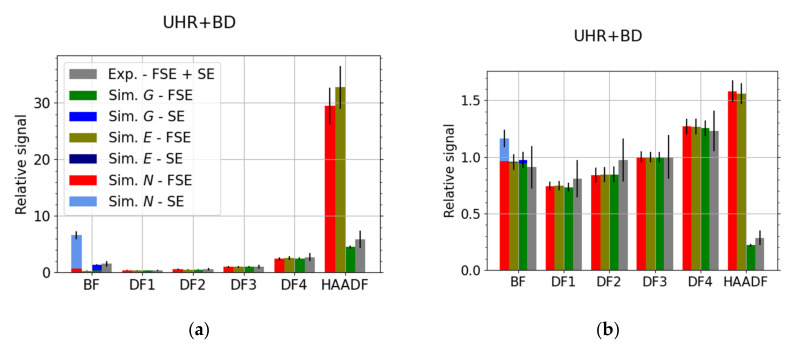
(**a**) Mo and (**b**) C; comparison of simulated and experimental data for UHR + BD microscope mode, *E_L_* = 15 keV. Simulation data at the best-match thickness contains all three (from left to right): electron counts *N*, total energy dose *E* and the calibrated (CC-based) total grayscale *G*. The data have been scaled to the segment DF3.

Let us briefly discuss the disadvantages of using electron counts *N* or energy dose, instead of the above provided CC-scaled energy dose in the relative comparison, i.e., scaled w.r.t. a selected segment. The resulting values are presented in Figure 10. The counts will fail to describe the situation properly if there is a mix of high energy and low energy electrons such as peaks on both sides of Figure 4. Not all electrons contribute with the same weight. This effect is strengthened in the BD mode where all electrons, including the secondary ones, are accelerated. Indeed, it is especially visible in the BF segment for the UHR + BD modes in Figure 10, more pronounced in the case of Mo where there are more (relatively speaking) SEs when compared to the carbon. The energy dose can be sufficient for description provided the response of individual parts of the detector (segmented or pixelated) is the same. We expect this to be true in the case of 2D-PAD but the Figure 2 makes clear this does not apply to the segmented STEM detector we have used. Indeed, the high values of the energy dose *E* or the counts *N* for HAADF segment in Figure 10 illustrate the discrepancy.

**Figure 11:**
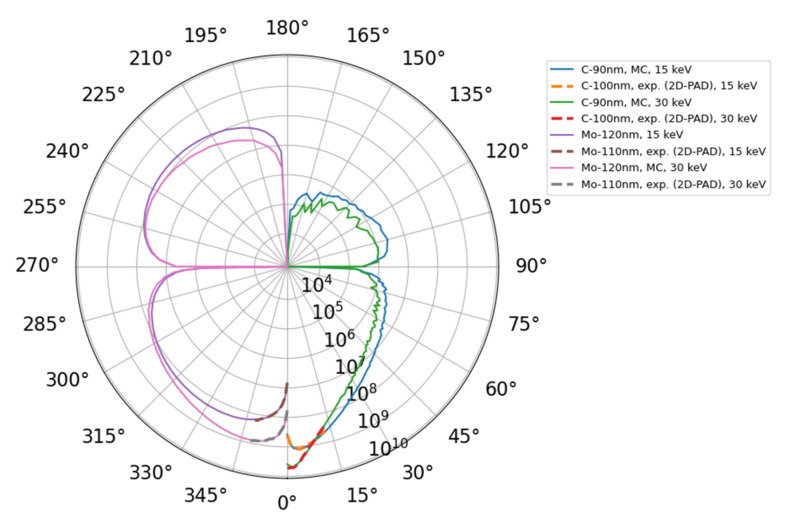
Polar plot of area-corrected and scaled experimental data (dashed lines) C 100 nm (right) and Mo 110 nm (left) for both 15 keV and 30 keV. The experimental data have been scaled to values of energy (solid lines) collected—within polar angle of step 1 deg—from MC simulations (10^6^ electrons). Best-match thickness, i.e., glassy-carbon 90 nm and Mo 120 nm, was used. Thus, the scale of the “radial” axis reflects the energy dosage in eV enclosed within each polar angle bin.

The scattering process (see Figure 8 and Figure 9) is best visualized using a logarithmic polar diagram. The simulated and experimental intensities are displayed in Figure 11 for both materials, C and Mo, and for both landing energies, 15 keV and 30 keV. We find nice agreement of the simulated and measured data for the restricted angular range-the 2D-PAD acceptance polar angle is about 13 degrees. The covered angular range can be extended using a larger 2D-PAD, these configurations are used as well.

Figure 11 well illustrates practical knowledge of microscopists that the maximum of scattered intensity shifts to higher angles with lower landing electron energy or for higher atomic numbers *Z*. This *Z* dependence is useful when interpreting micrographs of nanomaterials produced by BF vs DF. Figure 11 shows explicitly how the local maximum of intensity shifts to higher angles when changing the sample from carbon-based (low-atomic number) to molybdenum foil (high-atomic number). The just described composition sensitivity together with thickness dependence, illustrated in Figure 6, Figure 8 and Figure 9, in principle enable us to determine both composition and thickness. Moreover, this method could also be applied to assess thickness of an oxide layer.

To be more precise, the MC simulations used in this comparison depend on the combination of both material parameters (density and the parameters of both elastic and inelastic scattering) and thickness. Therefore, such estimates should be used with care.

Additional factors, such as diffraction and misalignment of the detector, may complicate the thickness estimation from segmented STEM detectors. The detailed information provided by the 2D-PAD helped us to find “center of the intensity” and to interpret and identify local maxima in Figure 8 as diffraction peaks. Let us note that there are other approaches to determine thickness using STEM, but their scope is often limited, e.g., STEM-defocus series applicable to crystalline materials only [55], see also references therein. The intensity increase, corresponding to diffraction, in both BF and DF1 segments makes clear that one should not normalize the data relative to segments close to the optical axis.

The diffraction peaks are clearly visible for C 100 nm thin foil at landing energy equal to 30 keV only, see Figure 8b. They seem not to be present at the lower value of landing energy, i.e., 15 keV displayed in Figure 8a. Thus, we speculate that graphite grains are present, especially at the bottom of the thin foil. Since the discrete diffraction spots arise from coherent scattering of the incident beam, see reference [56], and e.g., inelastic mean free path (IMFP) at 15 keV is approximately a half of the value at 30 keV, it does not come as a surprise that the diffraction is suppressed at the lower value of the landing energy. We compare the angles corresponding to the Debye–Scherrer diffraction rings of data displayed in Figure 8 with graphene (i.e., a “single layer graphite”) diffractogram [57] (Figure 6) and simple calculations using the Bragg formula (lattice constant *a* = 2.461 Å, interlayer distance *c* = 6.708 Å), see Table 1. We estimate the diffraction angle by using a single pair of opposite diffraction spots for each diffraction order, except for the lowest one, in the case of graphene. The overall agreement is good except for the lowest order of graphene where the read-out was unreliable since the diffraction spots were too close to the central spot of the primary beam.

The IMFP in molybdenum is smaller than IMFP of carbon, thus the diffraction is expected to be suppressed even more. Hence its apparent absence in the Mo 110 nm sample at both 15 and 30 keV; see Figure 9. We also analyzed 20 nm thin foil using the 2D-PAD at 30 keV; the nominal sample thickness is comparable with IMFP value. The diffraction was strong; therefore, the comparison with MC simulations would be meaningless and the sample was not analyzed any further. We know that our molybdenum sample preparation method may result into nano-crystallinity of the sample, thus diffraction was to be expected. A different sophisticated approach would have to be utilized as already mentioned in Section 2.4.

The diffraction ring diameter depends on a de Broglie wavelength, a lattice spacing in the crystal and it is proportional to an effective camera length. The actual value of camera length can be acquired using a calibration sample of known lattice spacing. Determined camera length value affects the subsequent processing of diffraction patterns and comparison with simulations. Knowledge of precise value is needed, e.g., if one intends to use diffraction patterns to calculate lattice spacing. This is not our goal, as we merely wish to present our calibration method for comparing experimental results with simulations. Therefore, we use construction values provided with the measuring devices. We have tested that a deviation 1 mm in camera length value would roughly correspond to offset 2 nm in the best-match thickness in the case of the 2D-PAD.

The previous discussion clarifies that the sample thickness is a key parameter and its independent measurement is desirable. We have already estimated the thickness for the Mo sample, using a profilometer, at a different region not transferred to the TEM grid. Another independent option is available. One can destroy the sample by FIB milling and directly measure the thickness of a “perpendicularly” oriented cut region of interest of the sample, where the previous experiments took place, in the electron microscope [58]. Because the results corresponding to the carbon based sample results indicate a good agreement with nominal value of sample thickness, well within the tolerance indicated by the provider, we did not use this destructive method. The sample thickness can also be determined using the convergent beam diffraction technique in TEM operating at high primary beam energies [59]. The thickness measurement by the pixelated detector takes place locally and therefore depends on the local inhomogeneity of the sample. In a low-energy STEM, the angular distribution of TEs gives information about the sample thickness [60]. Determination of sample thickness can be performed with low-energy HAADF [61]. Electron Energy Loss Spectroscopy (EELS) can provide another option to determine the thicknesses of the regions of interest in thin foils [56]. The data processing involved in some of the previously mentioned methods makes these approaches not truly independent. Conversely, if the thickness is known with sufficient precision, we can use the comparison method to test parameters of the MC simulations, namely the material density.

## 5. Conclusions

We have described the existing challenges in interpreting intensities, measured via segmented and pixelated detectors, beyond a qualitative approach. Both results of quantitative measurements complement each other. We compared them with MC simulations successfully, and the processing described in the previous sections clearly shows that each detector requires a different approach. The new calibration method for the segmented STEM detector presented in this paper was necessary to make the corresponding comparison feasible. Nevertheless, application of MC methods allowed us to estimate sample thickness from data representing an average over a selected region of interest, segmented detector, and from local measurement at the spot of the incident beam, 2D-PAD.

## Figures and Tables

**Table 1:** Comparison of diffraction angles (in degrees) for rings or spots for carbon-based materials measured at 30 keV. The experimental data are from C 100 nm thin foil diffraction maxima, Figure 8, and graphene diffractogram from reference [57] (Figure 6).

“Order”\Source	C 100 nm, 2D-PAD	Graphene	Bragg Formula (hkl)
“1st”	1.0	-	1.0 (100)
“2nd”	1.7	1.8	1.7 (110)
“3rd”	3.0	3.1	2.9 (300)

## Data Availability

Not applicable.
